# The Effects of Ethnically Congruent Music on Eye Movements and Food Choice—A Cross-Cultural Comparison between Danish and Chinese Consumers

**DOI:** 10.3390/foods9081109

**Published:** 2020-08-12

**Authors:** Danni Peng-Li, Raymond C. K. Chan, Derek V. Byrne, Qian Janice Wang

**Affiliations:** 1 Food Quality Perception and Society, iSENSE Lab, Department of Food Science, Aarhus University, 8200 Aarhus N, Denmark; derekv.byrne@food.au.dk (D.V.B.); qianjanice.wang@food.au.dk (Q.J.W.); 2 Neuropsychology and Applied Cognitive Neuroscience Laboratory, CAS Key Laboratory of Mental Health, Institute of Psychology, Chinese Academy of Sciences, Beijing 100101, China; rckchan@psych.ac.cn; 3 Sino-Danish Center for Education and Research, Beijing 100049, China; 4 Department of Psychology, University of Chinese Academy of Sciences, Beijing 100101, China

**Keywords:** consumer behavior, cross-culture, eye-tracking, food choice, musical fit, sensory marketing

## Abstract

Musical fit refers to the congruence between music and attributes of a food or product in context, which can prime consumer behavior through semantic networks in memory. The vast majority of research on this topic dealing with musical fit in a cultural context has thus far been limited to monocultural groups in field studies, where uncontrolled confounds can potentially influence the study outcome. To overcome these limitations, and in order to explore the effects of ethnically congruent music on visual attention and food choice across cultures, the present study recruited 199 participants from China (*n* = 98) and Denmark (*n* = 101) for an in-laboratory food choice paradigm with eye-tracking data collection. For each culture group, the study used a between-subject design with half of the participants listening to only instrumental “Eastern” music and the other half only listening to instrumental “Western” music, while both groups engaged in a food choice task involving “Eastern” and “Western” food. Chi-square tests revealed a clear ethnic congruency effect between music and food choice across culture, whereby Eastern (vs. Western) food was chosen more during the Eastern music condition, and Western (vs. Eastern) food was chosen more in the Western music condition. Furthermore, results from a generalized linear mixed model suggested that Chinese participants fixated more on Western (vs. Eastern) food when Western music was played, whereas Danish participants fixated more on Eastern (vs. Western) food when Eastern music was played. Interestingly, no such priming effects were found when participants listened to music from their own culture, suggesting that music-evoked visual attention may be culturally dependent. Collectively, our findings demonstrate that ambient music can have a significant impact on consumers’ explicit and implicit behaviors, while at the same time highlighting the importance of culture-specific sensory marketing applications in the global food industry.

## 1. Introduction

Sensory marketing can be defined as “marketing that engages the consumers’ senses and affects their perception, judgment and behavior” [1]. In the context of food, researchers and practitioners have for decades been using this idea to study and create optimal dining and retail atmospherics that facilitate consumption and sales [2,3]. Auditory contributions to these sensory nudges are becoming an increasingly popular topic of research [4,5]. In particular, literature on ambient, atmospheric, or in-store music has been robustly shown to influence a myriad of consumer behavior and perception [6,7,8,9]. These include manipulations of both simple (e.g., volume and tempo) and more complex (e.g., affective and genre) acoustic parameters [10,11,12,13,14], both of which can generate spontaneous feelings and physiological reactions, (“embodied meaning”), as well as cognitively prime contextual associations through semantic networks in memory (“referential meaning”) [15]. 

Musical fit encapsulates the connotation of referential meaning. Specifically, it refers to music that is congruent with and fits the attributes of the food or product in question. For example, Western classical music is stereotypically associated with sophistication and luxury and is congruent and appropriate with up-scale, expensive dining experiences, thus leading to higher monetary expenditure [16,17].

Arguably one of the most cited papers on this topic is a study by North and colleagues [18], who demonstrated that British consumers were in fact implicitly influenced by the ethnically congruent music played in the background in the supermarket. Particularly, they showed over a two-week period, that French wines outsold German wines when French music was played in the background, and vice versa when German music was played. Interestingly, when participants were asked afterwards about their wine purchases, 98% did not mention the music, suggesting that consumer choice and behavior do not follow traditional economic models of rational decision-making [19]. Instead of deliberate decision processes, some food choices are merely based on autonomic and emotional systems [20], which can be guided through sonic cues in the environment [21,22].

Besides the music itself, culture-related consumer differences are certainly influential on musical perception and its priming effects. In a large sample study including almost 3000 respondents from the US and China, Cowen and colleagues [23] discovered that some musical features from Chinese and Western music may universally evoke distinct subjective experiences, while others are culturally determined. However, the evidence of such influence of ethnically congruent music on food choice/behavior in culturally diverse samples is still lacking. The majority of studies conducted so far have been confined to a single culture group—commonly Western participants [11,18,24,25], which is by no means representative of the actual global consumer market. In contrast, one study focusing on only Asian participants (i.e., Chinese, Malaysian, and Indian) found an effect of musical fit on food choice when both food options were Asian (Malaysian or Indian); but when the food options presented were more distinctive (Western vs. Malaysian), participants simply choose Malaysian food irrespectively of the music condition [26]. Hence, preference (with Asian food) for at least Asian populations was a stronger driver of food choice than the music played. 

Collectively, these studies provide critical and ecologically valid evidence on musical fit and food choice, but there are still two main concerns. First, even though they include ethnically diverse food and music from the “East” and “West”, the participants recruited are still confined to either one of two cultural groups, but not both, therefore disregarding the continuously reported differences between these cultures [27,28,29,30]. Secondly, such real-life studies do not control for inconsistent environmental, social, and physiological factors affecting the outcome, nor the underlying cognitive and affective mechanisms that may at least partially explain how some ethnically congruent music can influence certain groups of consumers but not others.

For the former concern, simply including both Eastern and Western participants for the same study paradigm in a single study would circumvent this issue. For the latter concern, a number of implicit measures can be employed to achieve a more reliable result based on holistic procedures and not solely interpretation of choice/purchase data. These include biometric measurements such as galvanic skin response [31], facial expression [32], eye-tracking [33], etc. For instance, investigating eye movements in addition to choice data has been shown to be informative with respect to different factors of music [34], food [35], and culture [36]. Nevertheless, only one study thus far has been incorporating these three aspects together in a single study [37], yet not in the context of ethnic musical congruency. 

The present study therefore recruited both Chinese and Danish participants as representations of Eastern and Western consumers, respectively, for an eye-tracking choice paradigm with Eastern and Western food images and music excerpts. In line with previous musical fit literature, we expected that music’s cultural origin would draw attention to, and bias choice for, foods that are congruent with the music. Specifically, we hypothesized that

1. Eastern music would lead to more fixations on Eastern foods, and Western music would similarly lead to more fixations on Western foods.
2. These fixation patterns could congruently predict the choice of the food, consistent with the “gaze bias theory” of visual attention and choice [38].

Importantly, despite the previously reported cultural differences between Eastern and Western cultures in terms of musical priming effects [23], our previous study similarly including Chinese and Danish participants, but with taste-congruent soundtracks, did not find any major interaction effects between music played and culture in the fixation and choice analyses [37]. Therefore, we did not a priori expect any cultural dependency with regard to fixations nor food choice. However, another study (not incorporating music) did find that Chinese (vs. American) participants had marginally more revisits, i.e., they alternated their fixations more between the food and background when observing different food images with varying background saliency [36]. The authors suggested that such difference could be rooted in a generally more holistic (vs. analytic) world view in Chinese participants. Accordingly, we also hypothesized that

3. Chinese participants would have more revisits compared to Danish participants.

## 2. Materials and Methods 

### 2.1. Participants

A total of 199 participants (98 Chinese and 101 Danish) took part in the main study. Two participants from China and four from Denmark were removed from the analysis due to eye-tracking quality < 70%, resulting in a final sample size of 193 participants of whom 96 were Chinese (mean age ± SD = 22.16 ± 2.73 years; 65% females) and 97 who were Danish (mean age ± SD = 23.81 ± 2.85 years; 61% females). The Chinese participants were university students in Beijing, China, who were recruited through participant pools on the social media platforms WeChat and QQ. The Danish participants were college/university students in Aarhus, Denmark, who were recruited through flyers at the Aarhus University Campus and social media including student Facebook groups. Therefore, the study consisted of two identical experiments which were carried out in two different locations (Beijing, China and Aarhus, Denmark), but with equivalent physical environment and experimental setup (see Section 2.4). All participants fulfilled the screening criteria and reported having normal or corrected-to-normal hearing, and normal or corrected-to-normal vision without color blindness. Written informed consent was obtained from all participants. The study was approved by the Ethics Committee of Aarhus University (approval number: 2019-616-000019), and conducted in accordance with the ethical standards laid out in the Declaration of Helsinki and Ethics Committee of the Institute of Psychology, Chinese Academy of Sciences. All participants were compensated for their participation (Chinese participants were monetarily compensated via Alipay or WeChat pay; Danish participants were rewarded a gift card).

### 2.2. Visual Stimuli

**Figure 1:**
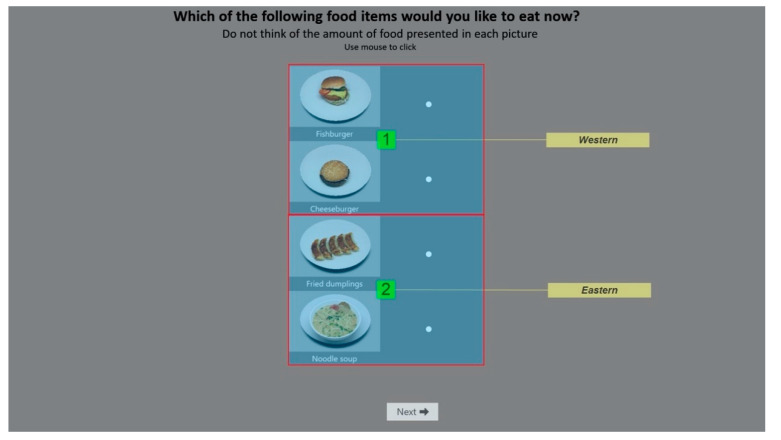
Example of a choice stimulus with two Western food items on the top and two Eastern food items on the bottom. Participants were instructed to choose one of the four food items that they wanted to eat the most using a mouse click. The overlaid labels indicated corresponds to the AOIs drawn out in the iMotions software ^®^ (not shown to participants).

High resolution, standardized food images included in the current study was obtained from the CROCUFID: A cross-cultural food image database [39]. Twenty food images were selected; ten typical Eastern food items (steamed meat buns, fried dumplings, fried rice, fried noodles, noodle soup, noodles with pork, pork over rice, fresh spring rolls, stir-fried vegetables, mapo tofu) and ten typical Western food items (chicken wrap, spicy chicken wrap, Greek salad, quinoa salad, risotto, pizza, grilled sandwich, cheeseburger, fish burger, mashed potatoes). These food items were used to construct twenty different “menus”/trials each containing four different food items (two Eastern and two Western) placed vertically on top of each other on a grey background [40]. The two food items from each culture were always placed together, and using Latin square randomization, each individual food items appeared four times (twice on top and twice on the bottom) on the trials (Figure 1). All food images can be found at: https://osf.io/5jtqx/ and have been validated in terms of their cultural origin (see Appendix A for image numbers; Table A1).

### 2.3. Auditory Stimuli

Two music excerpts were used in the study to represent “Eastern” and “Western” music, respectively. The Eastern music excerpt was from Hong Ting’s performance of Lotus out of water, a traditional Chinese composition; and the Western excerpt was an interpretation of the jazz standard Autumn leaves, by Tal Farlow. Both excerpts only included a single plucked string instrument, the Chinese guzheng and acoustic guitar. The tempo of the excerpts was normalized to 90 bpm on average using Audacity 2.3.2 for Mac OS version 16.33 and were played from a Bose SoundLink Revolve Bluetooth speaker (Årslev, Denmark) at approximately 55 decibels in order to mimic a more realistic setting with ambient music. We specifically chose not to use headsets as it would unnecessarily draw attention to the sound component of the study, and we did not want to distract participants from the food choice task. Previous studies have endorsed the applicability of speakers in experiments with food and music [37,41,42]. We therefore controlled for listening volume by ensuring that the Bluetooth speaker was always placed in the same point in the room with respect to the participant (i.e., 80 cm away). The excerpts can be heard at: https://soundcloud.com/danni-peng-li/sets/hongting-tal-farlow-90bpm.

### 2.4. Design and Procedure

**Figure 2:**
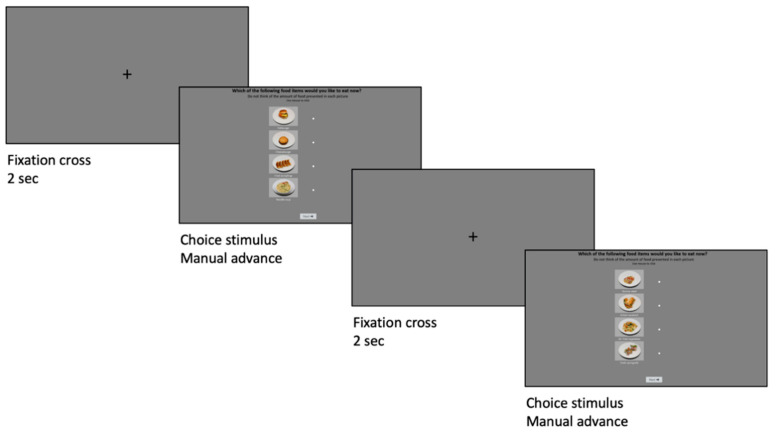
Example of two trials. Before each trial, a fixation cross is presented for 2 s before participants make a food choice while eye-tracking is recorded. The trials were randomized between participants.

Participants were asked to refrain from consuming food and drinks (except for water) two hours prior to the study, in order control for effects of hunger on food choice [43,44]. Participants entered the experiment room with artificial lightning (i.e., no sunlight) and seated approximately 70 cm from the HP EliteDisplay E243i, 24″, 16:10 monitor (screen resolution of 1920 × 1080 pixels; Årslev, Denmark, and Beijing, China). They were instructed to minimize head movements during eye-tracking recording. Before initiating the experiment, calibration of the screen-based Aurora Smart Eye eye-tracker (Årslev, Denmark), with a 60 Hz sampling rate and a head rotation and gaze accuracy of 0.3°, was done. The two sound conditions were divided between participants, such that half of the Chinese and Danish participants only listened to Eastern music, while the other half only listened to Western music. Music was played at the beginning of the calibration period and throughout the entire eye-tracking experiment of 20 trials. For each trial, they were shown a menu card (see Section 2.2) and instructed to choose one of the four food items that they wanted to eat in the given moment using a mouse click, while eye-tracking data was collected. No time constraint was set, but they were asked to choose simply based on their initial thought. To control for exposure bias, a fixation cross that participants had to focus on was displayed for 2 s before each trial (Figure 2).

**Figure A1:**
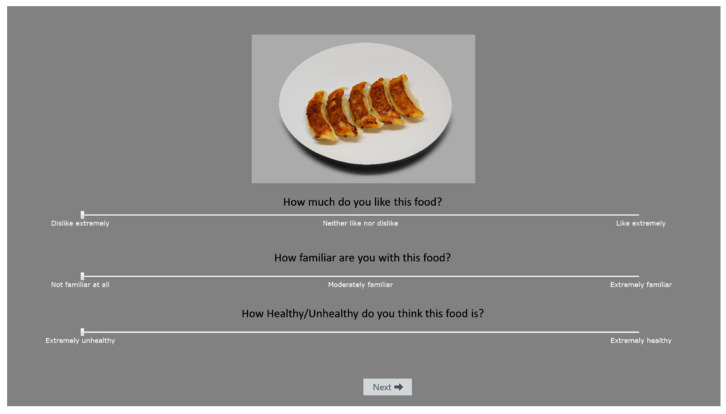
Example of a question in the follow-up questionnaire (liking, familiarity, and healthiness).

Participants underwent a training session of four trials prior to actual data collection. Based on the food categories, two areas of interest (AOI; Eastern AOI and Western AOI) were defined for each trial, as we were not particularly interested in each specific food item per se, but whether they were Eastern or Western (Figure 1). After choice paradigm with eye-tracking, participants answered a follow-up questionnaire in order to control for potential biases to the eye-tracking results. This included three questions about each of the twenty food items (liking: anchored with “Dislike extremely” to “Like extremely”; familiarity: anchored with “Not familiar at all” to “Extremely familiar”; and healthiness: anchored with “Extremely unhealthy” to “Extremely healthy”), with ratings given on continuous 9-point scales (see Appendix A; Figure A1). Likewise, participants listened to both music excerpts for 15 s followed by liking ratings and whether they believed them to be “Asian”, “Western”, or neither. The order of the food items shown and excerpt played were randomized for each participant. It took approximately 30 min for each participant to completely the whole study. The design of the experiment and preprocessing of data were carried out using iMotions^®^ software (https://imotions.com/platform/). 

### 2.5. Data Analysis

#### 2.5.1. Eye-Tracking Metrics

**Figure A2:**
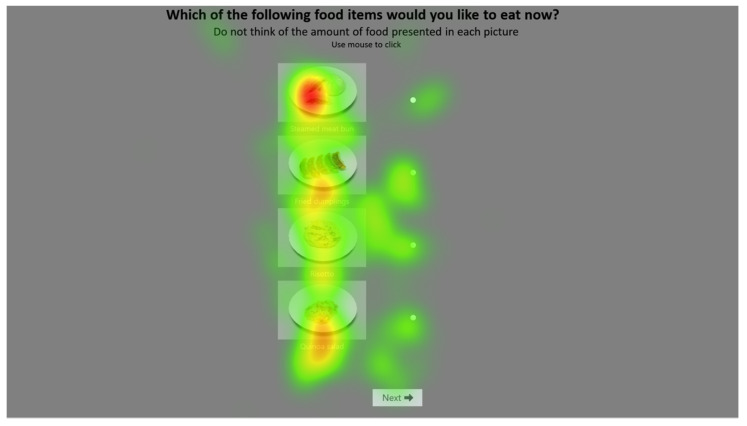
Heatmap of a random trial for a participant listening to Eastern music.

One gaze point equals to one raw sample captured by the eye-tracker. At a sampling rate of 60 Hz, each gaze point represents a sixtieth of a second (16.67 ms). A fixation denotes when a series of gaze points happens to be close in time and range, resulting in gaze cluster (i.e., when our eyes are locked towards a specific entity). The typical duration of a fixation is 100–300 ms. Total fixation time is based on the total duration of participants’ fixations (excludes datapoints between fixations). Fixation count represents number of fixations recorded within an AOI. Revisit count indicates the number of fixation returns/revisits to an AOI (i.e., if a participant fixates on an AOI, fixate on somewhere else, and then return to the same AOI). We initially focused on these three metrics as they have been widely used in eye-tracking research [45,46]. In the preprocessing, a Velocity-Threshold Identification (I-VT) fixation-filter was set and fixations slower than 60 ms were removed from the analysis. See Appendix A (Figure A2) for an example of and heatmap based on fixation values.

#### 2.5.2. Statistical Analysis

All data was organized in Microsoft Excel for Mac OS version 16.33, and the analyses were performed in RStudio for Mac OS version 1.3.959. 

Analysis of choice data was performed using chi-square independence tests to examine the relation between the music conditions (Eastern vs. Western) and food choice (Eastern vs. Western) for each culture. Yates’ continuity correction was applied to all chi-square tests. 

Eye-tracking data was analyzed using a generalized linear mixed models (GLMM) via the glmer()-function of the lme4 package in R. GLMM has been extensively employed in eye-tracking literature with repeated measures designs [47,48,49] as it depicts the response as a combination of fixed and random effects, and accounts for the hierarchical structure and non-independence of observations from individual participants by adding random intercepts to the models [50]. The models are very versatile due to their applicability to various types of variables: Gaussian (total fixation time), Poisson (fixation/revisit count), and binary (choice).

For the current eye-tracking analyses, the independent variables in the model were comprised of “culture” (between-subject factor; Chinese vs. Danish), “music condition” (between-subject factor; Eastern vs. Western), and “AOI” (Within-subject factor; Eastern vs. Western), which were coded as fixed effects. “Participant ID” and “Trial number” entered the model as random effects. The dependent variables of interest initially included Total fixation time, Fixation count, and Revisit count. Omnibus tests were carried out to test the main effects and interactions between the fixed independent variables. If a significant interaction was indicated by the GLMM, follow-up Bonferroni-corrected post hoc analyses were performed on the simple main effects using *t*-tests. These eye-tracking metrics were furthermore used in a multiple logistic regression as regressors on the binomial variable of food choice (chosen = 1; not chosen = 0), to assess the relationship between eye movement and eventual food choice, regardless of the food type. 

Follow-up questionnaires were analyzed using one-way multivariate analysis of variance (MANOVA) to assess culture-specific differences in regard to liking, familiarity, and healthiness ratings of the combined variables of Eastern food (based on the average score of the ten Eastern food items) and Western food (based on the average score of the ten Western food items), as well as liking of the two music excerpts. If a global effect were found, Bonferroni corrected univariate one-way analysis of variance (ANOVA) was then performed on each dependent variable. In addition, paired t-tests (2-tailed) were performed to test for within-culture differences in liking, familiarity, and healthiness ratings between Eastern vs. Western food and liking between Eastern vs. Western music.

## 3. Results

### 3.1. Choice 

**Figure 3:**
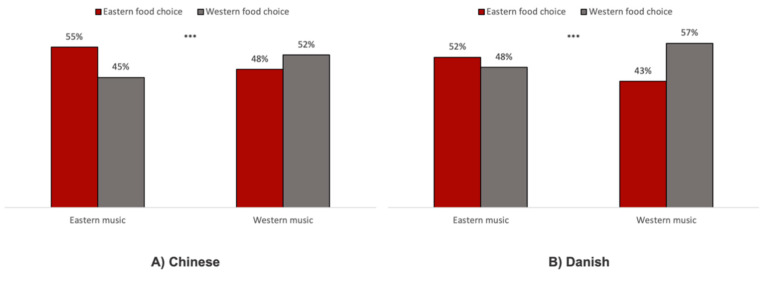
Choice distributions of (**A**) Chinese participants and (**B**) Danish participants, based on the chi-square tests of independence. *** indicates *p* < 0.001.

The chi-square tests of independence revealed that Chinese participants listening to Eastern (vs. Western) music choose significantly more Eastern (and less Western) food items (proportion_Eastern_music_ = 55.22% vs. proportion_Western_music_ = 47.51%; *χ*^2^_(1)_ = 10.94; *p* < 0.001; Figure 3A). Similarly, for Danish participants, Western (vs. Eastern) music induced significantly more Western (and less Eastern) food choices (proportion_Eastern_music_ = 48.28% vs. proportion_Western_music_ = 56.56%; *χ*^2^_(1)_ = 12.96; *p* < 0.001; Figure 3B).

### 3.2. Eye-Tracking

A summary of the omnibus eye-tracking results is shown in Table 1, which is further explained in this section. To avoid redundancy and for clearer visual interpretation of the differences between the two cultures (as the choice results), we focused on two reduced models on each level of culture in the subsequent post analyses; one for Chinese participants only and one for Danish participants, each with two independent variables (music condition and AOI). Furthermore, due the tendency of high correlation between total fixation time and fixation count [51], we performed a correlation analysis on these to variables and found that they indeed were highly correlated for both Chinese (*r* = 0.88; *t*_(3838)_ = 113; *p* < 0.001) and Danish (*r* = 0.87; *t*_(3878)_ = 111; *p* < 0.001) participants. Therefore, to circumvent statistical redundancy, we focused on fixation count (instead of total fixation time) and revisit count. The exclusion also evades potential multicollinearity in our regression model [52]. All post hoc analyses reported are Bonferroni corrected for multiple comparisons and explained in the following sections.

#### 3.2.1. Fixation Count—Chinese Participants 

**Figure 4:**
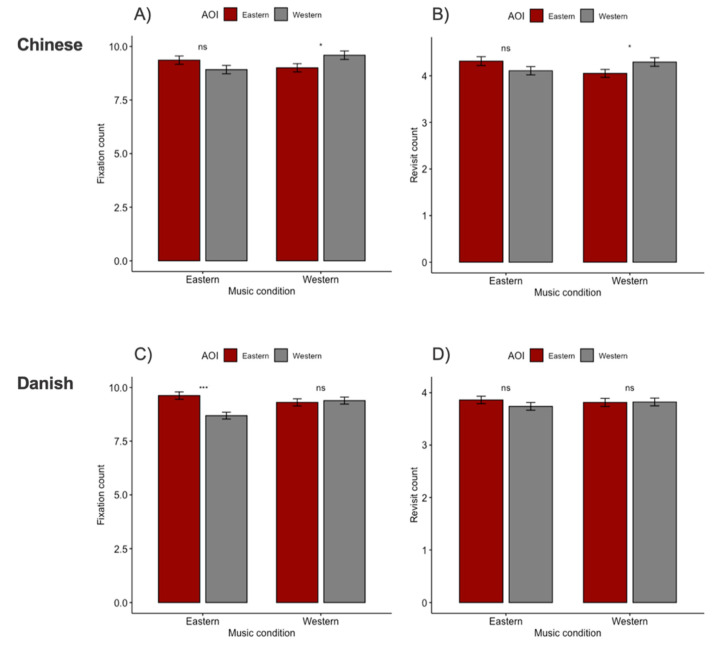
Barplots with standard error of post hoc analyses. Dark red denotes to Eastern food items/AOI and grey denotes Western food items/AOI for the two music conditions. Top two plots illustrate results of (**A**) fixation count and (**B**) revisit count for Chinese participants and bottom two plots show (**C**) fixation count and (**D**) revisit count for Danish participants. ns indicates no significance, * indicates *p* < 0.05, *** indicates *p* < 0.001.

For Chinese participants, an interaction effect in fixation count between music and AOI (*z*_(3834)_ = 5.25; *p* < 0.001; Table 1) was detected. Post hoc analyses on the simple main effects showed that participants listening to Western music fixated significantly more on Western (vs. Eastern) food items (*t*_(959)_ = 1.93; *p* = 0.022), but Eastern music did not lead to any differences (*t*_(959)_ = −2.55; *p* = 0.110; Figure 4A).

#### 3.2.2. Fixation Count—Danish Participants 

For Danish participants, an interaction effect in fixation count between music and AOI (*z*_(3874)_ = 5.26; *p* < 0.001; Table 1) was observed. Post hoc analyses on the simple main effects showed that participants listening to Eastern music had significantly more fixations on Eastern (vs. Western) food items (*t*_(1906)_ = 3.98; *p* < 0.001), but no differences were detected in the group only listening to Western music (*t*_(1956)_ = −0.36; *p* = 1.000; Figure 4C).

#### 3.2.3. Revisit Count—Chinese Participants 

For Chinese participants, an interaction effect between music condition and AOI was found (*z*_(3834)_ = 3.41; *p* < 0.001; Table 1). Post hoc analyses on the simple main effects indicated that participants listening to Western music revisited the Western (vs. Eastern) food significantly more (*t*_(959)_ = −2.35; *p* = 0.038), but no differences were observed in the participants listening to Eastern music (*t*_(959)_ = 1.89; *p* = 0.119; Figure 4B).

#### 3.2.4. Revisit Count—Danish Participants 

No main nor interaction effects were observed in the Danish participants in regard to revisit count (Table 1; Figure 4D). 

#### 3.2.5. Relationship between Food Choice and Eye Movements

The multiple logistic regression model for Chinese participants showed that the prediction variable of fixation count (*z*_(3834)_ = 17.87; *p* < 0.001) and revisit count (*z*_(3834)_ = -1.98; *p* = 0.048) contributed significantly to food choice (Table 2). Correspondingly, for Danish participants, both fixation count (*z*_(3834)_ = 18.84; *p* < 0.001) and revisit count (*z*_(3834)_ = -3.96; *p* < 0.001) could predict food choice. Fixation count was for both cultures the strongest predictor, meaning that the AOI that were fixated at more were correspondingly chosen more.

### 3.3. Follow-Up Questions

#### 3.3.1. Food Items

**Figure 5:**
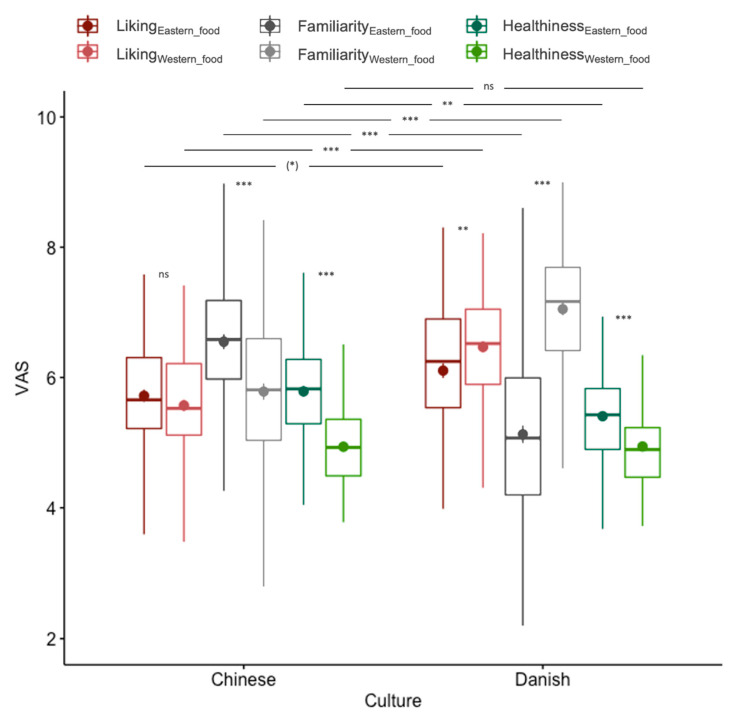
Boxblot of the liking, familiarity, and healthiness visual analogue scale (VAS) ratings of the Eastern vs. Western food items within and between cultures. ns indicates no significance, (*) indicates *p* < 0.1, ** indicates *p* < 0.01, *** indicates *p* < 0.001.

The MANOVA analysis (Figure 5) indicated a significant difference between the two cultures on the combined dependent variables (liking_Eastern_food_, liking_Western_food_, familiarity_Eastern_food_, familiarity_Western_food_, healthiness_Eastern_food_, healthiness_Western_food_; *F*_(1, 191)_ = 68.28; *p* < 0.001). Post hoc univariate ANOVA found that all dependent variables (except healthiness_Western_food_) contributed to the significant global effect after correcting for multiplicity. That is, Chinese participants rated both Eastern (*t*_(191)_ = −2.62; *p* = 0.056; trend) and Western (*t*_(191)_ = −7.30; *p* < 0.001) food significantly lower in terms of liking compared to Danish participants. 

In terms of familiarity, Chinese (vs. Danish) participants rated Eastern food to be more familiar (*t*_(191)_ = 7.98; *p* < 0.001), and conversely, Danish participants were more familiar with Western food compared to Chinese participants(*t*_(191)_ = 7.98; *p* < 0.001). With regard to healthiness ratings, Chinese participants rated Eastern food to be significantly healthier, compared to Danish participants (*t*_(191)_ = 3.35; *p* = 0.002), but no participant background-based difference was detected for Western food (*t*_(191)_ = -0.03; *p* = 1.000). 

We also analyzed the within-culture differences using paired *t*-tests (2-tailed). The analyses showed that there was no difference in liking between Eastern and Western food for Chinese participants (*t*_(95)_ = 1.57; *p* = 0.119); but for Danish participants, Western food was liked significantly more (*t*_(96)_ = -3.29; *p* = 0.001). For familiarity, Chinese participants were significantly more familiar with Eastern (vs. Western) food (*t*_(95)_ = 8.54; *p* < 0.001) and Danish were congruently more familiar with Western food (*t*_(96)_ = 15.69; *p* < 0.001). Finally, both Chinese (*t*_(95)_ = 10.67; *p* < 0.001) and Danish (*t*_(96)_ = 6.73; *p* < 0.001) participants rated Eastern food to be healthier than Western food.

#### 3.3.2. Music Excerpts

**Figure 6:**
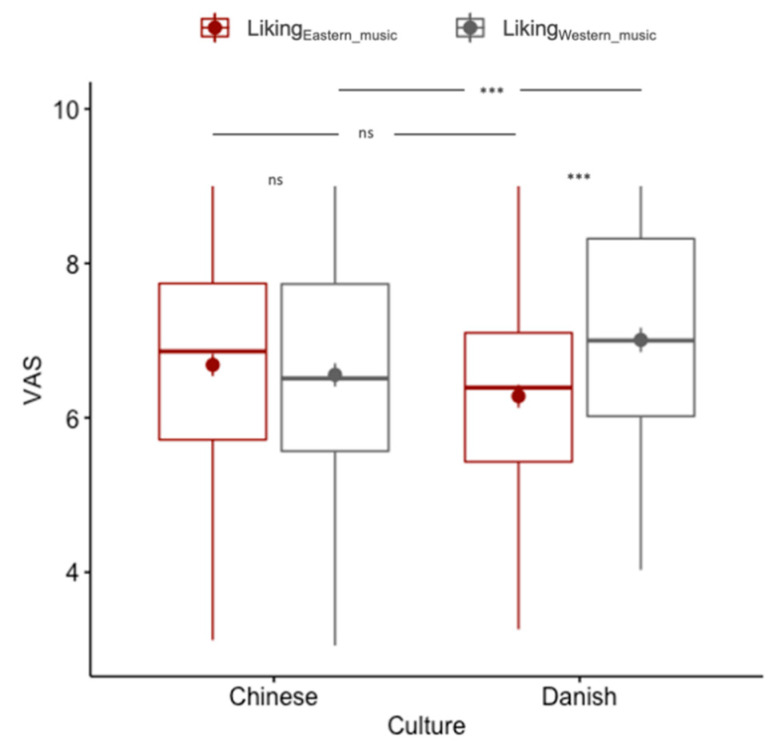
Boxplot of the liking ratings of the Eastern vs. Western music excerpts by Chinese and Danish participants. ns indicates no significance, *** indicates *p* < 0.001.

The MANOVA analysis (Figure 6) revealed a significant difference between the two cultures on the combined dependent variables (liking_Eastern_music_, liking_Western_music_; *F_(1_*_, *191)*_ = 4.94; *p* = 0.008). Post hoc univariate ANOVA found that the dependent variable, liking_Western_music_ contributed significantly to the global effect. Specifically, Danish participants liked the Western music significantly more than Chinese participants (*t_(191)_* = −7.30; *p* < 0.001), but no difference was observed with regard to Eastern music (*t_(191)_* = 1.93; *p* = 0.11). The within-culture paired *t*-tests (2-tailed) showed that there was no difference in liking between Eastern and Western music for Chinese participants (*t_(95)_* = −0.64; *p* = 0.263), but for Danish participants, Western (vs. Eastern) music was liked significantly more (*t_(96)_* = 4.00; *p* < 0.001). Finally, 97% and 85% of the participants categorized the Eastern excerpt to be Eastern and the Western excerpt to be Western, respectively.

## 4. Discussion

Although the ideas and applications of sensory marketing have been employed widely by practitioners from small- to large-scale businesses [2,53], and studies have explored the effects of musical fit on consumer behavior [11,25,26], this is the first study that focuses on the ethnic congruity between “East” and “West” in terms of participants, music, and food. By utilizing eye-tracking to quantify visual attention during a laboratory-based food choice task, our study has also circumvented the uncertainties of previous experiments that exclusively focused on the choice itself in uncontrolled environments. 

Consistent with our hypothesis 1, effects of musical fit on visual attention and food choice were observed. A clear congruent difference between what the participants chose and what they listened to was likewise reflected in the chi-square choice results. In line with previous literature [18,26], we found that both Chinese and Danish participants chose significantly more Eastern (vs. Western) food in the Eastern music condition and Western (vs. Eastern) food in the Western music condition. Not surprisingly, Chinese participants were more familiar with Eastern food items and Danish participants were more familiar with Western ones. 

Interestingly, and partially in contrast to our hypothesis 1, our results showed that participants’ fixations were only affected by the music which was ethnically different from their culture. That is, Chinese participants fixated more on Western food when Western music was played (but without difference for participants listening to Eastern music), whereas Danish participants fixated more on Eastern food when Eastern music was played (without any difference for participants in the Western music condition). The study by Yeoh and North [26] revealed that musical fit effects in Asian participants only occur when there was no clear preference between the competing foods. Our findings suggested, however, that the influence of musical fit effect on visual attention only occurred whenever the music was incongruent with participants ethnic culture, but regardless of food preference within the two cultures. In other words, we still observed priming effects for music that were different from participants’ own culture despite differences in food preference.

Importantly, in the domain of visual attention, these effects were more prominent when measuring fixation count rather than revisit count, as determined by lower *β* estimate and higher *p*-value in the regression analyses. In fact, fixation count was the strongest predictor of food choice, meaning for every unit of change in fixation count, the log odds of choice (vs. no choice) would increase by 0.25 and 0.26 for Chinese and Danish participants, respectively. Manippa and colleagues [54] similarly found that their visual primes did not influence the participants’ food choices, but dwell time and fixation count were higher for chosen versus non-chosen foods. Consequently, in line with our hypothesis 2, people simply look more at food that is eventually chosen, consistent with previous studies [55,56], confirming the “gaze bias theory” [38]. Notably, some evidence suggest that increased visual attention to a product does not always in practice imply it is chosen [57,58].

Put together, these results collectively suggest that ethnically congruent music can invariably impact consumer choice as well as visual attention in both Western and Eastern cultures, possibly by creating referential meaning through semantic connotations. On a broader level, our findings demonstrate that not only extensively studied extrinsic product factors such as packaging and label design [33,59], but also contextual, ambient stimuli unrelated to the product itself can influence consumers’ visual attention. 

In light of the ongoing discussion on the stereotypically reported cultural differences in human cognition, where “East” is more collectivistic and focuses on structural determinants of behavior, and the “West” relying more on analytical and individualistic processing and reasoning [60,61,62], none of our findings clearly reflected this view. Interestingly, concerning revisit count, only Chinese participants listening to Western music had more revisits to Western (vs. Eastern) food items, which is in line with our hypothesis 3. One may argue that this reflects Easterners’ more “holistic” viewing patterns as they alternate their fixations more between the AOI. Although there was a lack of significance, Zhang and Seo [36] also found that there was a trend for Chinese (vs. American) to have more revisits to the food from the background, and in particularly these increased as background saliency increased, while Americans tended to fixate more at the individual focal food objects. Hence, when background cues (whether it was table and plate decorations or music) are more salient or different from the norm (such as instrumental Western jazz), they may prompt more visually explorative behavior in individuals of Eastern ethnicity as previously reported [61,63].

On the other hand, these subtle differences may simply be due to the lower familiarity and higher palatability ratings of the Western food, and/or more hesitation of the choice. Besides, since Chinese participants fixated more on Western food in the Western music condition, even though there were no clear preference between the two music excerpts, the increased revisits are disputably just the product of musical fit and choice hesitation. That is, more revisits to Western food is likely manifested in predominantly deliberate “system 2” thinking as opposed to decisions based on more immediate “system 1” processes [64,65] when participants were exposed to Western music.

### Limitations

It should be noted that the affective and cognitive processes mentioned above are highly dependent on other components which were not accounted for in the current study. For instance, a limitation is that we did not control for personality traits nor BMI, but only included background info of age, gender, and culture. It has previously been documented that individuals with lower self-esteem, poorer foresight, and more impulsive behavior, as observed in e.g., obese participants, are more susceptible to be influenced by sensory nudges [20,66]. In contrast, a recent study revealed that visual nudging interventions were efficient regardless of impulsivity traits [67], suggesting that at least some forms of nudging effects are applicable consistently through the consumer spectrum. Nonetheless, a more refined stratification of groups based on individual differences in addition to cultural background would indeed improve the current study. 

Along the same lines, one may speculate that the demographic inclusion of “Chinese” participants is too broad a categorization, as there is enormous cultural variation within China itself [68]. However, our participants were only recruited from universities in Beijing, China, and have therefore lived in Beijing during at least their university years. In addition, the majority of Chinese citizens are still influenced by the same Confucian ideologies of collectivism and holism [69], and in any case, the within-China diversity is indisputably still smaller than the difference between China versus Denmark, or “East” versus “West”. 

That said, despite the similar demographic background (age and gender distribution) of Chinese and Danish participants, one should be aware of the generalizability of responses between these two groups due to differences in response style and conceptualization [70]. For example, we cannot know for sure that the subject ratings such as music liking reflected participants’ actual preference. Although there was reportedly no difference between the two cultures for the Eastern music, other implicit electrophysiological measures would have been able to validate these responses [71]. This could perhaps clarify whether or not the auditory stimuli used also generated embodied meaning (i.e., spontaneous feelings) through affective priming, and not solely referential meaning through semantic congruency and memory. In other words, we would be able to test if the music played also induced emotional and affective responses, in addition to the semantic congruency. Particularly, if one of the music excerpts were evidently more preferred over the other, this might have been a decisive factor captured by (neuro)physiological measures, and thereby leading to difference in reward valuation and emotional regulation as observed in a study by Salimpoor and colleagues [72], This could result in modified hedonic experience of the food items through so-called “sensation transference” [73] and consequently affect visual attention and eye-movements. 

In addition, we did not explicitly ask participants about familiarity of the music excerpts. Such information would add to the understanding about the referential meaning of the music retrieved from memory and prior experiences. Conversely, we expediently asked whether they believed the music to be categorized as either “Eastern” or “Western”, which at least confirmed the ethnic congruency by a majority of the participants.

Finally, our study included a terminological/technological uncertainty, namely the quantitative inference of visual attention. Although there is a there is a strong association between eye movements and attention [74], we must still concede that what is being fixated on does not always on its own imply that the object is perceptually or cognitively processed in brain [46]. In order to accommodate this limitation as best as possible, we therefore discarded fixations that were lower than 60 ms using the I-VT-fixation-filter during preprocessing [75].

## 5. Conclusions

The present study is the first to explore implicit behavioral effects of ethnically congruent music between Eastern and Western participants. Our findings illustrate that the notion of musical fit on visual attention and food choice is universal across cultures. However, the congruency bias is not particularly uniform for Chinese and Danish participants. Instead, the visual saliency towards specific food categories is arguably more prominent whenever the music excerpts are incongruent with one’s cultural background. In other words, the musical fit effect seems to work best with relatively unfamiliar food/music combinations. To increase the reliability and confidence to practitioners and stakeholders, future studies should aim to replicate these findings with various types of consumers and musical interventions [76]. Nevertheless, we have highlighted the importance of culture-specific ambient music and demonstrated that subtle but simple acoustic adjustments may have a significant impact on consumer behavior. On a broader level, practitioners at any commercial level should exploit the effects of sensory marketing and consider culturally tailored experiences, not only in modality of sound, but also in terms of the overall eating atmospherics [77]. In order to optimize their businesses, such auditory applications would in particular be relevant for marketers with international customer bases.

## Figures and Tables

**Table 1:** Summary of significant eye-tracking results of the fixed effects in the omnibus generalized linear mixed models (GLMM) tests. Post hoc analyses are not included in the table. Top part of the table includes the three-way model with both cultures. Below is each separate model for Chinese and Danish participants, respectively, which were focused on in the subsequent analyses.

	Fixation Count	Revisit Count
Chinese and Danish participants (*N* = 193)		
Culture	*p* > 0.100	*p* > 0.100
Music	*p* > 0.100	*p* > 0.100
AOI	*z*_(7710)_ = −3.20; *p* = 0.001 **	*z*_(7710)_ = −2.21; *p* = 0.027 *
Culture × Music	*p* > 0.100	*p* > 0.100
Culture × AOI	*z*_(7710)_ = −2.52; *p* = 0.012 *	*p* > 0.100
Music × AOI	*z*_(7710)_ = 5.25; *p* < 0.001 ***	*z*_(7710)_ = 3.41; *p* < 0.001 ***
Culture × Music × AOI	*p* > 0.100	*p* > 0.100
Chinese participants (*n* = 96)		
Music	*p* > 0.100	*p* > 0.100
AOI	*z*_(3834)_ = −3.20; *p =* 0.001 **	*z*_(3834)_ = −2.20; *p =* 0.027 *
Music × AOI	*z*_(3834)_ = 5.25; *p* < 0.001 ***	*z*_(3834)_ = 3.41; *p* < 0.001 ***
Danish participants (*n* = 97)		
Music	*p* > 0.100	*p* > 0.100
AOI	*z*_(3874)_ = 6.76; *p* < 0.001 ***	*p* > 0.100
Music × AOI	*z*_(3874)_ = 5.26; *p* < 0.001 ***	*p* > 0.100

* indicates *p* < 0.05, ** indicates *p* < 0.01, *** indicates *p* < 0.001.

**Table 2:** Model summary of multiple logistic regression with food choice (chosen = 1; not chosen = 0) as the binomial dependent variable, and fixation count, revisit count, and music condition as the independent variables.

	*β*	*S.E.*	Wald’s *z*	*df*	*p*	95% CI
Chinese participants (*n* = 96)						
						
(Intercept)	−2.07	0.12	−17.04	3834	<0.001	[−2.31–1.84]
Fixation count	0.25	0.01	17.87	3834	<0.001 ***	[0.22–0.28]
Revisit count	−0.05	0.02	−1.98	3834	0.048 *	[−0.10–0.01]
Danish participants (*n* = 97)						
(Intercept)	−2.01	0.11	−17.62	3874	<0.001	[−2.24–1.79]
Fixation count	0.26	0.01	18.83	3874	<0.001 ***	[0.23–0.29]
Revisit count	−0.10	0.03	−3.95	3874	<0.001 ***	[−0.15–0.05]

* indicates *p* < 0.05, *** indicates *p* < 0.001.

## Appendix A

**Table A1:** Table of food items included in the eye-tracking paradigm. Extracted from the CROCUFID: A cross-cultural food image database (https://osf.io/5jtqx/; Toet et al., 2019).

Food Item	Image Number	Food Category
Steamed meat buns	460	Eastern
Fried dumplings	519	Eastern
Fried rice	396	Eastern
Fried noodles	487	Eastern
Noodle soup	533	Eastern
Noodles with pork	422	Eastern
Pork over rice	526	Eastern
Fresh spring rolls	405	Eastern
Stir fried vegetables	524	Eastern
Mapo tofu	551	Eastern
Chicken wrap	666	Western
Spicy chicken wrap	124	Western
Greek salad	9	Western
Quinoa salad	668	Western
Risotto	105	Western
Grilled sandwich	95	Western
Pizza	357	Western
Cheeseburger	292	Western
Fishburger	8	Western
Mashed potatoes	352	Western

## References

[1] Krishna A. (2012). An integrative review of sensory marketing: Engaging the senses to affect perception, judgment and behavior. J. Consum. Psychol..

[2] Krishna A., Cian L., Sokolova T. (2016). The power of sensory marketing in advertising. Curr. Opin. Psychol..

[3] Spence C. (2012). Gastrophysics-The New Science of Eating.

[4] Seo H.-S. (2020). Sensory Nudges: The Influences of Environmental Contexts on Consumers’ Sensory Perception, Emotional Responses, and Behaviors toward Foods and Beverages. Foods.

[5] Spence C. (2020). Simple and complex crossmodal correspondences involving audition. Acoust. Sci. Technol..

[6] Kantono K., Hamid N., Shepherd D., Yoo M.J.Y., Carr B.T., Grazioli G. (2016). The effect of background music on food pleasantness ratings. Psychol. Music.

[7] Milliman R.E. (1986). The Influence of Background Music on the Behavior of Restaurant Patrons. J. Consum. Res..

[8] Poupis L.M. (2018). Wishful hearing: The effect of chronic dieting on auditory perceptual biases and eating behavior. Appetite.

[9] Wang Q.J., Woods A.T., Spence C. (2015). “What’s Your Taste in Music?” A Comparison of the Effectiveness of Various Soundscapes in Evoking Specific Tastes. Iperception..

[10] Milliman R.E. (1982). Using Background Music to Affect the Behavior of Supermarket Shoppers. J. Mark..

[11] Zellner D., Geller T., Lyons S., Pyper A., Riaz K. (2017). Ethnic congruence of music and food affects food selection but not liking. Food Qual. Prefer..

[12] Biswas D., Lund K., Szocs C. (2019). Sounds like a healthy retail atmospheric strategy: Effects of ambient music and background noise on food sales. J. Acad. Mark. Sci..

[13] Reinoso-Carvalho F., Gunn L., Molina G., Narumi T., Spence C., Suzuki Y., Horst E., Wagemans J. (2020). A sprinkle of emotions vs a pinch of crossmodality: Towards globally meaningful sonic seasoning strategies for enhanced multisensory tasting experiences. J. Bus. Res..

[14] Mathiesen S.L., Mielby L.A., Byrne D.V., Wang Q.J. (2020). A systematic investigation of the relative importance of tempo and articulation on eating time. Appetite.

[15] Zhu R., Meyers-Levy J. (2005). Distinguishing between the Meanings of Music: When Background Music Affects Product Perceptions. J. Mark. Res..

[16] Areni C.S., Kim D. (1993). The Influence of Background Music on Shopping Behavior: Classical Versus Top-Forty Music in a Wine Store. Adv. Consum. Res..

[17] North A.C., Shilcock A., Hargreaves D.J. (2003). The Effect of Musical Style on Restaurant Customers’ Spending. Environ. Behav..

[18] North A.C., Hargreaves D.J., McKendrick J. (1999). Influence of in-store music on wine selection. J. Appl. Psychol..

[19] Rangel A., Camerer C., Montague P.R. (2008). A framework for studying the neurobiology of value-based decision making. Nat. Rev. Neurosci..

[20] Chen R., Peng-Li D., Turel O., Sørensen T.A., Bechara A., Li Y., He Q. (2018). Decision Making Deficits in Relation to Food Cues Influence Obesity: A Triadic Neural Model of Problematic Eating. Front. Psychiatry.

[21] Knoferle K.M., Spangenberg E.R., Herrmann A., Landwehr J.R. (2012). It is all in the mix: The interactive effect of music tempo and mode on in-store sales. Mark. Lett..

[22] Helmefalk M., Hultén B. (2017). Multi-sensory congruent cues in designing retail store atmosphere: Effects on shoppers’ emotions and purchase behavior. J. Retail. Consum. Serv..

[23] Cowen A.S., Fang X., Sauter D., Keltner D. (2020). What music makes us feel: At least 13 dimensions organize subjective experiences associated with music across different cultures. Proc. Natl. Acad. Sci. USA.

[24] North A.C., Sheridan L.P., Areni C.S. (2016). Music Congruity Effects on Product Memory, Perception, and Choice. J. Retail..

[25] Herget A.-K., Breves P., Schramm H. (2020). The Influence of Different Levels of Musical Fit on the Efficiency of Audio-Visual Advertising. Music. Sci..

[26] Yeoh J.P.S., North A.C. (2010). The effects of musical fit on choice between two competing foods. Music. Sci..

[27] Chiu L.-H. (1972). A Cross-Cultural Comparison of Cognitive Styles in Chinese and American Children. Int. J. Psychol..

[28] Goh J.O., Tan J.C., Park D.C. (2009). Culture Modulates Eye-Movements to Visual Novelty. PLoS ONE.

[29] Shankar M.U., Levitan C.A., Spence C. (2010). Grape expectations: The role of cognitive influences in color–flavor interactions. Conscious. Cogn..

[30] Zhang Q., Elsweiler D., Trattner C. (2020). Visual Cultural Biases in Food Classification. Foods.

[31] Kantono K., Hamid N., Shepherd D., Lin Y.H.T., Skiredj S., Carr B.T. (2019). Emotional and electrophysiological measures correlate to flavour perception in the presence of music. Physiol. Behav..

[32] Gunaratne T.M., Fuentes S., Gunaratne N.M., Torrico D.D., Gonzalez Viejo C., Dunshea F.R. (2019). Physiological Responses to Basic Tastes for Sensory Evaluation of Chocolate Using Biometric Techniques. Foods.

[33] Piqueras-Fiszman B., Velasco C., Salgado-Montejo A., Spence C. (2013). Using combined eye tracking and word association in order to assess novel packaging solutions: A case study involving jam jars. Food Qual. Prefer..

[34] Day R.-F., Lin C.-H., Huang W.-H., Chuang S.-H. (2009). Effects of music tempo and task difficulty on multi-attribute decision-making: An eye-tracking approach. Comput. Hum. Behav..

[35] Motoki K., Saito T., Nouchi R., Kawashima R., Sugiura M. (2018). Tastiness but not healthfulness captures automatic visual attention: Preliminary evidence from an eye-tracking study. Food Qual. Prefer..

[36] Zhang B., Seo H.-S. (2015). Visual attention toward food-item images can vary as a function of background saliency and culture: An eye-tracking study. Food Qual. Prefer..

[37] Peng-Li D., Byrne D.V., Chan R.C.K., Wang Q.J. (2020). The influence of taste-congruent soundtracks on visual attention and food choice: A cross-cultural eye-tracking study in Chinese and Danish consumers. Food Qual. Prefer..

[38] Schotter E.R., Berry R.W., McKenzie C.R.M., Rayner K. (2010). Gaze bias: Selective encoding and liking effects. Vis. Cogn..

[39] Toet A., Kaneko D., de Kruijf I., Ushiama S., van Schaik M.G., Brouwer A.-M., Kallen V., van Erp J.B.F. (2019). CROCUFID: A Cross-Cultural Food Image Database for Research on Food Elicited Affective Responses. Front. Psychol..

[40] Majaranta P., Aoki H., Donegan M., Hansen D.W., Hansen J.P., Hyrskykari A., Räihä K.-J. (2012). Gaze Interaction and Applications of Eye Tracking.

[41] Kontukoski M., Luomala H., Mesz B., Sigman M., Trevisan M., Rotola-Pukkila M., Hopia A.I. (2015). Sweet and sour: Music and taste associations. Nutr. Food Sci..

[42] Wang Q.J., Spence C. (2015). Assessing the Effect of Musical Congruency on Wine Tasting in a Live Performance Setting. Iperception.

[43] Duerlund M., Andersen B.V., Alexi N., Peng M., Byrne D.V. (2020). Subjective Sensations related to Food as Determinants of Snack Choice. Foods.

[44] Frank S., Laharnar N., Kullmann S., Veit R., Canova C., Hegner Y.L., Fritsche A., Preissl H. (2010). Processing of food pictures: Influence of hunger, gender and calorie content. Brain Res..

[45] iMotions® Eye Tracking The Complete Pocket Guide. https://imotions.com/blog/eye-tracking/.

[46] Orquin J.L., Loose S.M. (2013). Attention and choice: A review on eye movements in decision making. Acta Psychol. (Amst.).

[47] Kliegl R. (2007). Toward a perceptual-span theory of distributed processing in reading: A reply to Rayner, Pollatsek, Drieghe, Slattery, and Reichle. J. Exp. Psychol. Gen..

[48] Hohenstein S., Matuschek H., Kliegl R. (2017). Linked linear mixed models: A joint analysis of fixation locations and fixation durations in natural reading. Psychon. Bull. Rev..

[49] Yan M., Zhou W., Shu H., Yusupu R., Miao D., Krügel A., Kliegl R. (2014). Eye movements guided by morphological structure: Evidence from the Uighur language. Cognition.

[50] Orquin J., Holmqvist K. (2019). A Primer on Eye Tracking Methodology for Behavioral Sciences. A Handbook of Process Tracing Methods.

[51] Orquin J.L., Holmqvist K. (2018). Threats to the validity of eye-movement research in psychology. Behav. Res. Methods.

[52] Midi H., Sarkar S.K., Rana S. (2010). Collinearity diagnostics of binary logistic regression model. J. Interdiscip. Math..

[53] Spence C., Puccinelli N.M., Grewal D., Roggeveen A.L. (2014). Store Atmospherics: A Multisensory Perspective. Psychol. Mark..

[54] Manippa V., van der Laan L.N., Brancucci A., Smeets P.A.M. (2019). Health body priming and food choice: An eye tracking study. Food Qual. Prefer..

[55] Gidlöf K., Dewhurst R., Holmqvist K., Wallin A. (2013). Using Eye Tracking to Trace a Cognitive Process: Gaze Behaviour During Decision Making in a Natural Environment. J. Eye Mov. Res..

[56] Peschel A.O., Orquin J.L., Mueller Loose S. (2019). Increasing consumers’ attention capture and food choice through bottom-up effects. Appetite.

[57] Van der Laan L.N., Hooge I.T.C., de Ridder D.T.D., Viergever M.A., Smeets P.A.M. (2015). Do you like what you see? The role of first fixation and total fixation duration in consumer choice. Food Qual. Prefer..

[58] Wang E., Cakmak Y.O., Peng M. (2018). Eating with eyes—Comparing eye movements and food choices between overweight and lean individuals in a real-life buffet setting. Appetite.

[59] Reale S., Flint S.W. (2016). The Impact of Menu Label Design on Visual Attention, Food Choice and Recognition: An Eye Tracking Study. J. Sens. Stud..

[60] Chua H.F., Boland J.E., Nisbett R.E. (2005). From The Cover: Cultural variation in eye movements during scene perception. Proc. Natl. Acad. Sci. USA.

[61] Masuda T., Wang H., Ishii K., Ito K. (2012). Do surrounding figures’ emotions affect judgment of the target figure’s emotion? Comparing the eye-movement patterns of European Canadians, Asian Canadians, Asian international students, and Japanese. Front. Integr. Neurosci..

[62] Barrett H.C. (2020). Towards a Cognitive Science of the Human: Cross-Cultural Approaches and Their Urgency. Trends Cogn. Sci..

[63] Nisbett R.E., Miyamoto Y. (2005). The influence of culture: Holistic versus analytic perception. Trends Cogn. Sci..

[64] Evans J.S.B.T. (2007). On the resolution of conflict in dual process theories of reasoning. Think. Reason..

[65] Peng-Li D., Sørensen T.A., Li Y., He Q. Lower structural brain connectivity in individuals with elevated food addiction symptoms. Appetite.

[66] Hunter J.A., Hollands G.J., Couturier D.-L., Marteau T.M. (2018). Effect of snack-food proximity on intake in general population samples with higher and lower cognitive resource. Appetite.

[67] Marques I.C.F., Ting M., Cedillo-mart D., Federico J.A.P. (2020). Effect of Impulsivity Traits on Food Choice within a Nudging Intervention. Nutrients..

[68] Zhou J.X., Arnold M.J., Pereira A., Yu J. (2010). Chinese consumer decision-making styles: A comparison between the coastal and inland regions. J. Bus. Res..

[69] Qian S. (2020). Chinese Culture.

[70] Ares G. (2018). Methodological issues in cross-cultural sensory and consumer research. Food Qual. Prefer..

[71] Xu Y., Hamid N., Shepherd D., Kantono K., Reay S., Martinez G., Spence C. (2019). Background soundscapes influence the perception of ice-cream as indexed by electrophysiological measures. Food Res. Int..

[72] Salimpoor V.N., Benovoy M., Larcher K., Dagher A., Zatorre R.J. (2011). Anatomically distinct dopamine release during anticipation and experience of peak emotion to music. Nat. Neurosci..

[73] Spence C., Gallace A. (2011). Multisensory design: Reaching out to touch the consumer. Psychol. Mark..

[74] Just M.A., Carpenter P.A. (1976). Eye fixations and cognitive processes. Cogn. Psychol..

[75] Olsen A. The Tobii I-VT Fixation Filter-Algorithm Description. http://www.vinis.co.kr/ivt_filter.pdf.

[76] Pashler H., Wagenmakers E. (2012). Editors’ Introduction to the Special Section on Replicability in Psychological Science. Perspect. Psychol. Sci..

[77] Fanelli R.M., Di Nocera A. (2018). Customer perceptions of Japanese foods in Italy. J. Ethn. Foods.

